# Primary malignant mesothelioma of the diaphragm with liver invasion: A case report and review of literature: Erratum

**DOI:** 10.1097/MD.0000000000015667

**Published:** 2019-05-13

In the article, “Primary malignant mesothelioma of the diaphragm with liver invasion: A case report and review of literature”,^[1]^ which appeared in Volume 98, Issue 15 of *Medicine*, Dr. Wen-Jing Huang's name appears incorrectly as Jing-Wen Huang.

## References

[1] Huang J-W Li Z-H Wang Z Primary malignant mesothelioma of the diaphragm with liver invasion: A case report and review of literature. *Medicine*. 2019 98:e15147. 30985689 10.1097/MD.0000000000015147 PMC6485870

